# Complex post-traumatic stress disorder and dissociation: Co-occurrence and relationships with depressive symptoms and functional impairments

**DOI:** 10.1192/j.eurpsy.2025.1200

**Published:** 2025-08-26

**Authors:** H. W. Fung, A. K. C. Chau, C. H. O. Huang, C. T. Y. Cheung, S. K. K. Lam

**Affiliations:** 1 School of Nursing, The Hong Kong Polytechnic University, Kowloon; 2 Department of Psychiatry; 3 Department of Social Work and Social Administration, The University of Hong Kong, Pokfulam; 4 Department of Social Work, Hong Kong Baptist University, Kowloon; 5 The Nethersole School of Nursing, The Chinese University of Hong Kong, Shatin, Hong Kong

## Abstract

**Introduction:**

Although complex post-traumatic stress disorder (CPTSD) is closely associated with dissociative symptoms, and although dissociation is often conceptualized as a response to trauma, less is known about the co-occurrence of dissociation and (C)PTSD.

**Objectives:**

This study examined the co-occurrence of CPTSD and dissociation and their relationship with depressive symptoms and functional impairments.

**Methods:**

We analyzed baseline data from a clinical trial that evaluated a web-based trauma program. This study included 220 treatment seekers from Hong Kong (Mage = 35.3; SD = 7.9; 82.3% female). Participants completed measures of CPTSD, dissociation, depression and functional impairment, which were assessed with the International Trauma Questionnaire (ITQ), the Dissociative Experiences Scale-Taxon (DES-T), the Patient Health Questionnaire-9, and the Work and Social Adjustment Scale, respectively.

**Results:**

In this sample, 34.1% screened positive for CPTSD only, 10.0% for dissociative symptoms only (DES-T ≥ 25), and 25.0% for both conditions. For participants with CPTSD (n = 130), 42.3% had dissociative symptoms. For participants with dissociative symptoms (n = 77), 7.8% had PTSD, and 71.4% had CPTSD. Hierarchical multiple regression analyses showed that, after controlling for age, gender, education level, and trauma exposure, PTSD (β = .127, p = .033), disturbances in self-organization (DSO) (β = .524, p < .001), and dissociative (β = .169, p = .002) symptoms were all associated with depressive symptoms. Yet, only DSO symptoms were associated with functional impairments (β = .519, p < .001).

**Conclusions:**

This study provides updated data regarding the co-occurrence of CPTSD/PTSD and dissociation. About half of people with CPTSD have considerable dissociative symptoms, while most dissociative individuals may be suffering from CPTSD/PTSD. Moreover, this study found that, among different trauma-related symptoms, DSO symptoms are the strongest predictor of depressive symptoms and functional impairments, highlighting the importance of providing skills training for trauma survivors to address these difficulties in clinical settings.

**Disclosure of Interest:**

None Declared

